# Integrated *in vitro* and *in silico* assessment of antimicrobial, chemical antioxidant, and cytotoxic activities of endophytic *Aspergillus* sp. GK1 isolated from *Mentha spicata* roots

**DOI:** 10.3389/fphar.2026.1937405

**Published:** 2026-10-16

**Authors:** Fahad Almarshadi, Walid Alesefir, Gaurav Kumar

**Affiliations:** 1 Public Health Department, College of Public Health and Health Informatics, University of Hail, Hail, Saudi Arabia; 2 Medical and Diagnostic Research Center, University of Hail, Hail, Saudi Arabia; 3 Internal Medicine Department, College of Medicine, University of Hail, Hail, Saudi Arabia; 4 Amity Institute of Microbial Technology, Amity University Rajasthan, Jaipur, Rajasthan, India

**Keywords:** ADMET analyses, antimicrobial, antioxidant, cytotoxic properties, docking studies, endophytic fungi, GC-MS analysis

## Abstract

**Introduction:**

Medicinal plants are a source of structurally diverse endophytic fungi which harbor significant pharmaceutical potential.

**Methods:**

In the present research, the antimicrobial, chemical antioxidant, and cytotoxic properties of the ethyl acetate extract of endophytic *Aspergillus* sp. GK1 isolated from *Mentha spicata* roots were evaluated using both *in vitro* and *in silico* assessments. The crude extract was subjected to GC–MS profiling, which tentatively identified several metabolites, followed by molecular docking, drug-likeness, pharmacokinetic and toxicity prediction analyses.

**Results and Discussion:**

The crude extract has shown broad spectrum antibacterial activity with inhibition zone of 15.66 ± 0.57 mm against *Escherichia coli*, 14.66 ± 1.52 mm against *Staphylococcus aureus*, 14.00 ± 0.00 mm against *Staphylococcus epidermidis* and 13.33 ± 0.57 mm against *Bacillus subtilis*. Minimum inhibitory concentration values were 16, 32, 64, and 64 μg/mL, while minimum bactericidal concentration values were 32, 32, 64, and 128 μg/mL, respectively. The extract showed concentration-dependent responses in chemical antioxidant assays, including DPPH radical-scavenging, hydrogen peroxide-scavenging, reducing-power and total antioxidant-capacity assays. A strong correlation between chemical antioxidant capacity and total phenolics (R2 = 0.9752–0.9986) and total flavonoids (R2 = 0.9814–0.9956) content was found. The extract exhibited concentration-dependent cytotoxicity against MCF-7 cells with an IC50 value of 69.20 μg/mL that had positive correlation with the phenolics and flavonoids content. The analysis of GC–MS points to the presence of several metabolites, including 2-Buten-1-one, 1,3-diphenyl- (dypnone, a β-methyl2-Buten-1-one, 1,3-diphenyl-; CID 95329) which showed the highest binding affinity to selected protein targets, along with favourable drug-likeness, in silico pharmacokinetic and toxicity properties. The extract demonstrated antibacterial activity, measurable chemical antioxidant/ radical-scavenging properties, and cytotoxic effects under the experimental conditions. Integrated experimental and computational studies highlighted the tentatively identified 2-Buten-1-one, 1,3-diphenyl- as a compound of interest for further investigation.

**Conclusion:**

These findings provide preliminary indication for the possible biological activity of the *Aspergillus* sp. GK1 extract and support further bioassay- guided fractionation, isolation, structural characterization, mechanistic studies, and in vivo analysis of its active constituents.

## 1 Introduction

Microbial infections remain a major and ongoing threat to global public health and are made even more acute by the swift spread and emergence of multi-drug resistant (MDR) pathogen strains. Antibiotic resistance is mainly a consequence of the overuse of antimicrobial drugs in clinical care, non-compliance with the prescribed treatment course, and inadequate infection control. As a result of this phenomenon, there is a dramatic increase in morbidity and mortality worldwide and modern medicine has an almost empty arsenal of effective treatment. AMR has caused ∼1.14 million deaths in 2021, and contributed to nearly 4.71 million deaths worldwide, is estimated to cause up to 1.91 million deaths per year by 2050 (Naghavi et al., 2024). AMR not only has clinical implications, it also has an economic cost, including the burden of healthcare expenditure and losses to the global GDP, with the annual GDP shortfall estimated to exceed $1 trillion after 2030 (World Bank, 2017). A worldwide collaboration of scientists and policymakers is necessary to solve this urgent health challenge by focusing on the rational use of available antimicrobials, on monitoring resistant strains with stringent epidemiological surveillance and on the search of structurally novel antimicrobial scaffolds from untapped natural sources.

In addition to the risk of drug resistance, cancer is a major cause of death and disease in humans, significantly affecting life expectancy and the healthcare system worldwide. Global Cancer Observatory 2022 estimates that 19.98 million new cancer cases and 9.74 million cancer deaths reported worldwide in 2022. The top five global cancer causes including lung, colorectal, liver, breast and stomach cancer, continue to cause most cancer cases and deaths. Among these, breast cancer is the second most commonly diagnosed cancer, with an estimated 2.30 million new cases worldwide in 2022, and the fourth leading cause of cancer-related deaths, with an estimated 666,103 deaths worldwide in 2022 (Bray et al., 2024). While the basic clinical tools of modern oncology (systemic chemotherapy, ionizing radiotherapy and surgical resection) continue to play the primary and most important role in the management of cancer, they are limited by high economic cost, systemic toxicity, and the inevitable emergence of cellular drug resistance. Thus, the need for new, effective and safe therapeutic agents for cancer prevention and treatment is a necessary requirement.

Free radical-induced oxidative stress is a well-known biochemical link between both infectious diseases and oncogenesis, and is a major mechanism for the initiation and clinical course of a variety of human diseases. Reactive oxygen species (ROS) are important signaling molecules which, however, when produced in excess, overwhelm the native antioxidant defense mechanisms of the cell. This oxidative imbalance causes significant damage to macromolecules, such as the peroxidation of membrane lipids, modification of functional proteins and the formation of harmful strand breaks in genomic DNA (Pooja et al., 2025; Sadiq, 2023). Therefore, the discovery of new natural molecules that are able to effectively remove free radicals and neutralize the oxidative stress is a good approach to cellular balance and to reduce the development of chronic degenerative diseases (Engwa et al., 2022).

With the aim of identifying new molecules capable of inhibiting microorganisms, cancer and oxidative stress, researchers are increasingly investigating the plant microenvironment, focusing on endophytic fungi. The endophytes are diverse microorganisms residing in the host plant for their entire life cycle or for a particular phase without inducing any immediate disease symptoms. Fungal endophytes are the most structurally diverse and biologically valuable group from biotechnological point of view, with predominantly fungi from the Ascomycota and Basidiomycota divisions, and the presence of a few phyla of bacteria and actinobacteria (Rodriguez et al., 2009; Schouten, 2019).

Fungal endophytes are highly significant biomedical resources with enormous application potential in the medical, agricultural and cosmetic sectors as they possess a very wide range of secondary metabolites such as alkaloids, flavonoids, phenolic acids, terpenoids, quinones, tannins, saponins and steroids with evolutionary potential to synthesize these compounds (Hashem et al., 2023; Singh et al., 2021). A long evolutionary history of continuous genetic exchange and metabolic co-evolution between endophyte and the host plant has allowed these fungi to synthesize plant-like chemicals over the long term. In recent past a large number of studies have reported the cytotoxic and anticancer effect of fungal endophytes in various cell lines and *in vivo* models (Ibrahim et al., 2018; Magagula et al., 2025; Clark-Pérez et al., 2024). Similarly studies have reported the strong, broad spectrum antimicrobial activity of endophytic fungi actions a variety of microorganisms (Du et al., 2020; Mendieta-Brito et al., 2025). Additionally, endophytic fungi have been found to possess high antioxidant capabilities in different assays (El-Sayed et al., 2024; Jia et al., 2025).

The study of endophytes traditionally has been directed towards common and widely distributed genera of fungi while characterization of the localized endophytic community associated with medicinal herbs is a critical step in the quest for new therapeutics. In particular, the genus *Aspergillus* is a prolific but also highly complex metabolic engine that has the ability to produce chemically unique, dense frameworks with an amazing medicinal property (El-Hawary et al., 2020). *Mentha spicata* L. Spearmint is an excellent host plant for this study as a famous traditional medicinal plant valued for its essential oil production, high antioxidant property and rich ethnobotanical values in the treatment of inflammatory and infectious diseases (Mahendran et al., 2021). The root-associated endophytes of *M. spicata* are a one of a kind chemical frontier that has the potential to discover functional biomolecules because the root systems of aromatic plants have highly competitive and specialized microenvironments.

Despite the growing interest in endophytic fungi as sources of novel bioactive metabolites, the biological and chemical potential of root-associated endophytic fungi from *M. spicata* remains poorly explored. In particular, limited information is available on the integrated evaluation of the antibacterial, chemical antioxidant, cytotoxic, and metabolite profiles of endophytic *Aspergillus* species associated with this medicinal plant. Moreover, studies combining experimental bioactivity screening with computational approaches to prioritize potential lead molecules from these endophytes are scarce. To address this knowledge gap, the present study aimed to isolate and characterize an endophytic fungi from the roots of *M. spicata* and evaluate its crude extract for antibacterial, chemical antioxidant, cytotoxic properties, GC–MS profiling, and exploratory computational analyses. Collectively, this study provides insights into the different properties of the crude extract of *Aspergillus* sp. GK1, with particular emphasis on the *in silico* interactions of its tentatively identified metabolites with selected antibacterial, cancer-associated, and oxidative stress-associated protein targets. These findings provide a basis for future bioassay-guided isolation, target-specific mechanistic investigations, and *in vivo* experimentation of the identified metabolites and their potential therapeutic relevance.

## 2 Materials and methods

### 2.1 Plant sample collection

*M. spicata* L. was selected as the host plant for the isolation of endophytic fungi. The plant material was obtained from an agricultural land in Jaipur, Rajasthan, India and identified as *M. spicata* L. based on its characteristic morphological features and local cultivation history. The roots were collected and processed under aseptic conditions for the isolation of endophytic fungi.

### 2.2 Isolation of endophytic fungi from the plant sample

The roots of the herbaceous plants selected for study were cleaned by washing in tap water and rinsed in distilled water to eliminate any debris from the surface of the roots. To surface sterilize the roots, they were submersed within 70% ethanol for 30 s, submersed within a solution of 5% sodium hypochlorite for 1 min, and once again submersed in 70% ethanol for 30 s all while being agitated. Following surface sterilization, the roots were air dried on sterile filter paper for a short time. After the roots were cut and placed on Potato Dextrose Agar (PDA) plates and incubated at 28 °C ± 2 °C for 3–5 days. Once the fungal growth is observable, the fungal sample was subculture onto PDA plates and incubated at 28 °C for 3–5 days to achieve a pure culture of fungi (Wang et al., 2025). Once cultures were purified, they were stored for preservation at 4 °C.

### 2.3 Identification of isolated fungi

Fungal isolate was characterized by a combination of morphological, microscopic and molecular features. Cultural characteristics were recorded on PDA plates and microscopic examination was done by using a lacto phenol cotton blue stain. The identification of the molecular level was achieved by sequencing the ITS region. The fungal DNA was extracted and subjected to ITS sequencing. ITS1 (Forward: TCC​GTA​GGT​GAA​CCT​GCG) and ITS4 (Reverse: TCC​TCC​GCT​TAT​TGA​TAT​GC) were used to amplify the ITS region by polymerase chain reaction (PCR). The PCR product was purified and sequenced by using the BDT v3.1 Cycle Sequencing Kit on an ABI 3500xl Genetic Analyzer. The sequence that was obtained was subjected to BLAST searching of the NCBI GenBank database and the most closely related sequences were chosen. Multiple sequence alignment was used to align the sequences using the following multiple sequence alignment tools and MEGA-X software was used to construct the phylogenetic tree (Wang et al., 2026).

### 2.4 Extraction of fungal metabolites

The fungal culture was inoculated in a 500 mL sterilized Potato Dextrose Broth. The culture was incubated in stationary position at 28 °C for 7 days. After incubation, the fungal mat was removed and the fermented broth was filtered through Whatman No. 1 filter paper and collected in sterilized bottle. The resultant filtrate was employed to recover the secondary metabolites by liquid–liquid extraction using ethyl acetate (1:1). The solvent phase was recovered and concentrated using a rotary evaporator and air-dried to yield a crude extract, which was stored under refrigeration (Makky et al., 2021).

### 2.5 Evaluation of biological properties

#### 2.5.1 Antimicrobial properties of the endophytic fungi

Bacteria cultures of *E. coli*, *Staphylococcus aureus*, *Staphylococcus epidermidis*, and *Bacillus subtilis* were obtained from our laboratory which were previously isolated from the clinical samples and identified using a combination of Colony characters, microscopic characters and biochemical tests. An antibiogram profile of the test bacteria was also determined using disc diffusion method. Data related to characterization and antibiogram is given in Supplementary Tables S1 and S2 respectively.

The crude fungal extract was dissolved in dimethyl sulfoxide (DMSO) to final concentration of 1 mg/mL and sterilized by passing through syringe filter with 0.45 µm membrane. The antibacterial activity was determined by using agar well diffusion method on Mueller–Hinton agar (MHA). The fresh bacterial cultures were standardized to 0.5 McFarland and spread with the help of sterile cotton swabs on MHA plates. Three wells (6 mm diameter) were aseptically punched into each agar plate using a sterile gel borer. After that, 100 µL of the fungal extract (1 mg/mL) was placed in the first well while 100 µL of DMSO was placed in the second well as a negative control and 100 µL of gentamicin (100 μg/mL) was placed in the third well as a positive control. Plates were then incubated at 37 °C for 24 h and the diameters of the zones of inhibition were measured. Each experiment was conducted three times and the results presented as mean ± SD (CLSI, 2016).

#### 2.5.2 Minimum inhibitory concentration (MIC) and minimum bactericidal concentration (MBC)

The MIC was determined by the broth microdilution test in sterile 96-well microtiter plates. Fungal extract was serially diluted in Mueller–Hinton broth to the final concentrations of 1–1,024 μg/mL. Extract solutions were made double the concentration (2–2,048 μg/mL) and then diluted with an equal volume of bacterial suspension to compensate for the additional bacterial suspension added later. Bacterial suspensions were adjusted to 0.5 McFarland standards and from the standardized suspension; 0.1 mL was transferred into 9.9 mL of freshly prepared double-strength MHB to obtain a 1:100 dilution. Following this extract dilutions (100 μL) were then added to each well along with 100 μL of bacterial suspension for a total volume of 200 μL per well. After 24 h of incubation at 37 °C, the MIC was determined as the minimum concentration that did not exhibit any visible growth.

To determine the MBC, 10 µL aliquots from wells that did not show any visible growth were streaked onto MHA plates and incubated at 37 °C for 24 h. The MBC was determined as the lowest concentration which completely prevented formation of bacterial colonies. To achieve reproducibility and reliability of the results, all experiments were performed in triplicate (CLSI, 2016; EUCAST, European Committee for Antimicrobial Susceptibility Testing of the European Society of Clinical Microbiology and Infectious Diseases, 2003).

### 2.6 Chemical antioxidant properties of the endophytic fungi

Chemical antioxidant properties of the endophytic fungal extracts, was evaluated through DPPH (1,1-diphenyl-2-picrylhydrazyl) radical scavenging assay (Nuerxiati et al., 2019), reducing power assay (Kan et al., 2015), hydrogen peroxide scavenging assay (Yadav et al., 2014) and total antioxidant capacity assays (Hifney et al., 2017). All the experiments were performed in dose dependent manner and conducted in triplicates.

### 2.7 Analysis of polyphenolic content

To ascertain polyphenolic content, total phenolic content (TPC) and total flavonoid content (TFC) were measured using the process as described in our previous paper (Jangid et al., 2025). TPC was measured by using standard Folin–Ciocalteu reagent method and the results were demonstrated in micrograms gallic acid equivalence (GAE). TFC was conducted by aluminum chloride method and the results were demonstrated in micrograms quercetin equivalent. Both the experiments were performed in dose dependant assay and conducted in triplicates.

### 2.8 Cytotoxic activity of endophytic fungi

The human breast adenocarcinoma (MCF-7) cell line was used to assess the cytotoxic effect of the endophytic fungi using the 3-(4,5-dimethylthiazol-2-yl)-2,5-diphenyltetrazolium bromide (MTT) assay. The crude fungal extract was dissolved in dimethyl sulfoxide to create a stock solution of 10 mg/mL and later diluted in Dulbecco’s Modified Eagle Medium to make the final concentration of crude fungal extract range between 5 and 80 μg/mL. The positive control used was chlorpromazine. The MCF-7 cell line was procured from the National Centre for Cell Science (NCCS), Pune, India and cultured in Dulbecco’s Modified Eagle Medium (DMEM) with 10% heat-inactivated fetal bovine serum (FBS), penicillin (100 IU/mL), streptomycin (100 μg/mL) and amphotericin B (5 μg/mL) under a humidified atmosphere containing 5% CO_2_ at 37 °C. Cells were harvested using Trypsin/EDTA solution, counted at 1 × 10^5^ cells/mL and seeded into sterile 96-well microplates (100 µL each well). The medium was changed after 24 h of incubation for cell attachment and the cells were incubated for 72 h with fresh medium and different concentrations of the fungal extract. The cellular morphology was observed under the microscope at 24 h intervals. After the incubation period, the culture medium was discarded and 50 µL of MTT solution (prepared in phosphate-buffered saline) was placed in each well and incubated for 3 h at 37 °C. The formazan crystals thus formed were dissolved using propanol (100 µL) and the absorbance measured at 540 nm on a microplate reader. The percentage cytotoxicity and cell viability were calculated from the untreated control cells and half-maximal inhibitory concentration (IC_50_) value was obtained from the dose response curve. Each experiment was repeated three times and the data obtained were presented as mean ± SD (Ramadan et al., 2025).

The percentage cytotoxicity was computed using the formula:

Percentage cytotoxicity = [(Ac - At)/Ac] × 100.

The absorbance of the sample is called as At, and the absorbance of the control is called as Ac.

### 2.9 Gas chromatography mass spectrometry analysis (GC-MS)

The bioactive volatile compounds in the crude fungal extract were identified using the GC-MS system and a capillary column (30 m × 0.25 mm internal diameter × 0.25 µm film thickness). Helium (99.99% purity) was used as a purge flow of 3.0 mL/min and carrier gas at 1.01 mL/min. The system was run under a pressure, 58.4 kPa, a linear gas velocity of 36.7 cm/s and an equilibrium time of 0.5 min. The injector and interface temperatures were kept at 250 °C and 290 °C, respectively. The oven temperature was first set to 60 °C for 4 min before being programmed to 280 °C at 8 °C per min, and held at this temperature until the end of the analysis. The fungal extract was dissolved in methanol to make it 1 mg/mL before injection. The mass spectra were acquired in the range between 50 and 650 mass to charge (m/z) with a total run time of 32.5 min. The mass spectra acquired were compared with the National Institute of Standards and Technology (NIST) 2017 Mass Spectral Library (APAWLY-9781119376743) for identification of the compounds (Kaur et al., 2020).

The relevant information on the host plant material, its processing, isolation and identification of the endophytic fungus, preparation of the fungal extract, and chemical characterization of the extract compounds is reported with reference to the applicable recommendations of the ConPhyMP guidelines (Supplementary Material: ConPhyMP Tables 1 and 2) (Heinrich et al., 2022).

**TABLE 1:** Antimicrobial activity of crude fungal extract prepared from *Aspergillus* sp. isolate GK1 against clinical bacterial isolates.

Test bacteria	Zone of inhibition (mm) (Mean ± SD, n = 3)		
	Fungal extract (1 mg/mL)	Positive control	Negative control (DMSO)
*S. aureus*	14.66 ± 1.52	23.33 ± 0.57	0 ± 0
*B. subtilis*	13.33 ± 0.57	22.0 ± 1.0	0 ± 0
*S. epidermidis*	14.0 ± 0.0	26.0 ± 0.0	0 ± 0
*E. coli*	15.66 ± 0.57	24.66 ± 0.57	0 ± 0

Positive Control: Gentamicin at 100 μg/mL

zone of inhibition is also including well diameter (6 mm).

**TABLE 2:** GC-MS analysis of ethyl acetate crude extract of *Aspergillus* sp. isolate GK1 isolated from the roots of *M. spicata.*

RT	Tentative compound	Formula	MW	Chemical class	CID
8.37 min	Octanoic acid, 2-hexyl-	C_14_H_28_O_2_	228	Branched-chain saturated fatty acid	109035
13.6	3-Isopropyl-1-methyl-4-methylamino-pyrrole-2,5-dione	C_9_H_14_O_2_N_2_	182	Pyrrole derivative (nitrogen-containing heterocycle)	597587
17.7	N-Hexadecanoic acid (Palmitic acid)	C_16_H_32_O_2_	256	Saturated fatty acid	985
18.3	2-Buten-1-one, 1,3-diphenyl-	C_16_H_14_O	222	Chalcone derivative (dypnone; β-methylchalcone)	95329
19.4	α-Linolenic acid	C_18_H_30_O_2_	278	Polyunsaturated fatty acid	5280934

### 2.10 Molecular docking analysis

#### 2.10.1 Ligand preparation

The compounds, which were determined by Gas Chromatography Mass Spectrometry (GCMS) analysis of the endophytic fungal extract was chosen as ligands in molecular docking experiments. The three-dimensional structures of the identified compounds were obtained from the PubChem database in Structure Data File (SDF) format. Ligand structures were loaded into UCSF Chimera, energy-minimized to get stable conformations and then changed into PDB format. Lastly, the ligands were optimally prepared in PDBQT format using AutoDock Tools with the addition of hydrogen atoms and Gasteiger charges (Pettersen et al., 2004).

#### 2.10.2 Receptor preparation

Protein Data Bank (PDB) was searched to retrieve the crystal structures of the antibacterial, cancer-associated, and oxidative stress-associated protein targets. The receptors that were chosen were the following: Epidermal Growth Factor Receptor (EGFR) Tyrosine Kinase (PDB ID: 1M17), Breast Cancer Susceptibility Protein 1 (BRCA1; PDB ID: 1JNX), Human Peroxiredoxin 5 (PDB ID: 1HD2), water-forming NAD(P)H oxidase from *Lactobacillus sanfranciscensis* (PDB ID: 2CDU), Penicillin-Binding Protein (PBP; PDB ID: 3VSL), and DNA Gyrase B (PDB ID: 6F86). EGFR and BRCA1 are key molecular drivers in breast cancer proliferation, survival, and DNA repair pathways, Peroxiredoxin 5 is a human antioxidant enzyme involved in peroxide detoxification, while the bacterial NAD(P)H oxidase (2CDU) was included as a model FAD-dependent oxidoreductase involved in oxygen reduction while DNA Gyrase & PBPs are essential, highly conserved bacterial targets involved in DNA replication and cell wall synthesis, respectively, covering both Gram-positive and Gram-negative antibacterial mechanisms. UCSF Chimera was used to prepare protein by eliminating co-crystallized ligands, water molecules, and other non-essential heteroatoms. Before saving the prepared protein structures in PDB format, polar hydrogen atoms were included and suitable atomic charges given. The receptor files were then translated into PDBQT to be docked (Berman et al., 2000).

#### 2.10.3 Molecular docking procedure

AutoDock Vina was used to conduct molecular docking simulations to assess the binding interactions between the chosen fungal metabolite and the target proteins. Blind docking was performed with a grid box covering the entire receptor structure (grid centre and dimensions are provided in Supplementary Table S3), so that no binding site was pre-defined. Docking was run in AutoDock Vina with exhaustiveness = [8]. The default AutoDock Vina parameters were run to perform docking calculations and several binding conformations were generated per ligand. The docking results were ordered based on the binding affinity (kcal/mol) they would predict and the conformation with the lowest binding energy was chosen as the most desirable binding conformation to analyze further (Trott and Olson, 2010). A blind approach was chosen deliberately, as an unbiased test of whether the ligands preferentially recognise functional binding sites rather than arbitrary surface regions. This exploratory screen was intended only to generate hypotheses and not to establish target engagement.

#### 2.10.4 Visualization and analysis of protein–ligand interactions

The docked protein–ligand complexes were visualized using PyMOL and UCSF Chimera. To investigate the three-dimensional orientation of the ligands in the receptor binding pocket, PyMOL was utilized and structural inspection of the docking poses was done using UCSF Chimera. The ligand-amino acid residue interactions, as well as hydrogen bonding and hydrophobic interactions, were evaluated to determine the binding properties and possible biological implication of the identified fungal metabolite (Pettersen et al., 2004; Rigsby and Parker, 2016).

### 2.11 ADMET analysis

The computationally prioritized compound, CID 95329, was chosen to undergo ADMET analysis due to its high binding affinity with all antibacterial, cancer-associated, and oxidative stress-associated protein targets. The pkCSM online server was used to predict the pharmacokinetic and toxicity properties. The canonical SMILES of CID 95329, found in the PubChem database was inputted into pkCSM to assess its ADMET profile, which offers information about its drug-likeness and predicted pharmacokinetic and toxicity characteristics for compound prioritization (Pires et al., 2015).

### 2.12 Statistical analysis

Experiments of antimicrobial, antioxidant, polyphenolic, cytotoxicity testing were performed in triplicate and values are shown as mean ± standard deviation. For statically significant differences between groups, one-way analysis of variance (ANOVA) followed by Tukey’s *post hoc* test was applied, and the significance level was set at P < 0.05. Statistical analysis was performed in MS Excel 2007 and GraphPad Prism 8.

## 3 Results and discussion

Endophytic fungi have a symbiotic relationship with the medicinal plants and have a significant influence on their microenvironment. These endophytic fungi are known for their ability to assist plants under stress and protect them from infections; they are also metabolic factories of unique chemical structures that are generated with high efficiency. The process of extracting medicinal substances directly from slow growing plants can take a long time, consume a lot of resources and be detrimental to the environment. Therefore, application of the rapidly growing secondary metabolites of the microbial endophytes provides a very fast, sustainable, and easily scalable way of modern drug discovery (Hashem et al., 2023).

In line with this, the current study investigates the biological and chemical characteristics of the endophytic fungus, *Aspergillus* sp. isolate GK1 which was successfully isolated from the root tissues of the medicinal plant *M. spicata*. A dual *in vitro* and *in silico* approach was used to evaluate the biomedical profile of this endophyte. It was comprehensively tested for its natural antioxidant, antimicrobial and cytotoxic activities using different *in vitro* assays. These tests were followed with GC-MS analysis to tentatively identify the main active constituents in the crude extract. The identified structures were then computationally docked and subjected to ADMET simulations to predict their potential binding interactions and pharmacokinetic properties. This study will use functional biological testing and chemical profiling strategies with advanced computing models to elucidate the multifaceted biological and chemical characteristics of *Aspergillus* sp. isolate, GK1. This will allow a valuable insight into the strategies and methods of microbial drug discovery and will strengthen the importance of root-associated endophytes as potential sources for natural active compounds. The results of the experimental tests and of the theoretical considerations are given below.

### 3.1 Isolation and characterization of endophytic fungi

The isolate of fungus grown on PDA showed a high rate of growth and was able to spread to the edge of the Petri dish and cover the surface with a thick aerial mycelial mat. The colony surface was white to yellowish, but quickly became more distinct, dark brown to a jet-black color, with dense conidiogenesis, forming a powdery and granular appearance (Figure 1B). Distinct morphological features characteristic of the genus *Aspergillus* were observed under a microscope using a LPCB wet mount preparation such as conidiophores, vesicle and radiating chains of globose, dark brown to black, rough walled conidial spores were observed (Figure 1C). To further support the morphological identification, molecular characterization was performed using ITS sequencing. The endophytic fungal isolate was assigned to *Aspergillus* sp. and designated as isolate GK1 based on ITS sequence analysis. The sequence was deposited in the NCBI GenBank database under accession number PZ502934.1. Phylogenetic analysis based on the ITS sequence (Figure 1D) placed the isolate within the black *Aspergillus* group, supporting its assignment to the genus *Aspergillus*. However, ITS-based identification alone does not provide sufficient resolution for definitive species-level identification within the black *Aspergillus* group. Accordingly, the isolate was conservatively designated as *Aspergillus* sp. isolate GK1 in the present study. Definitive species-level identification would require multilocus sequencing using additional taxonomic markers, such as calmodulin (CaM), β-tubulin (BenA), and/or RPB2.

**FIGURE 1:**
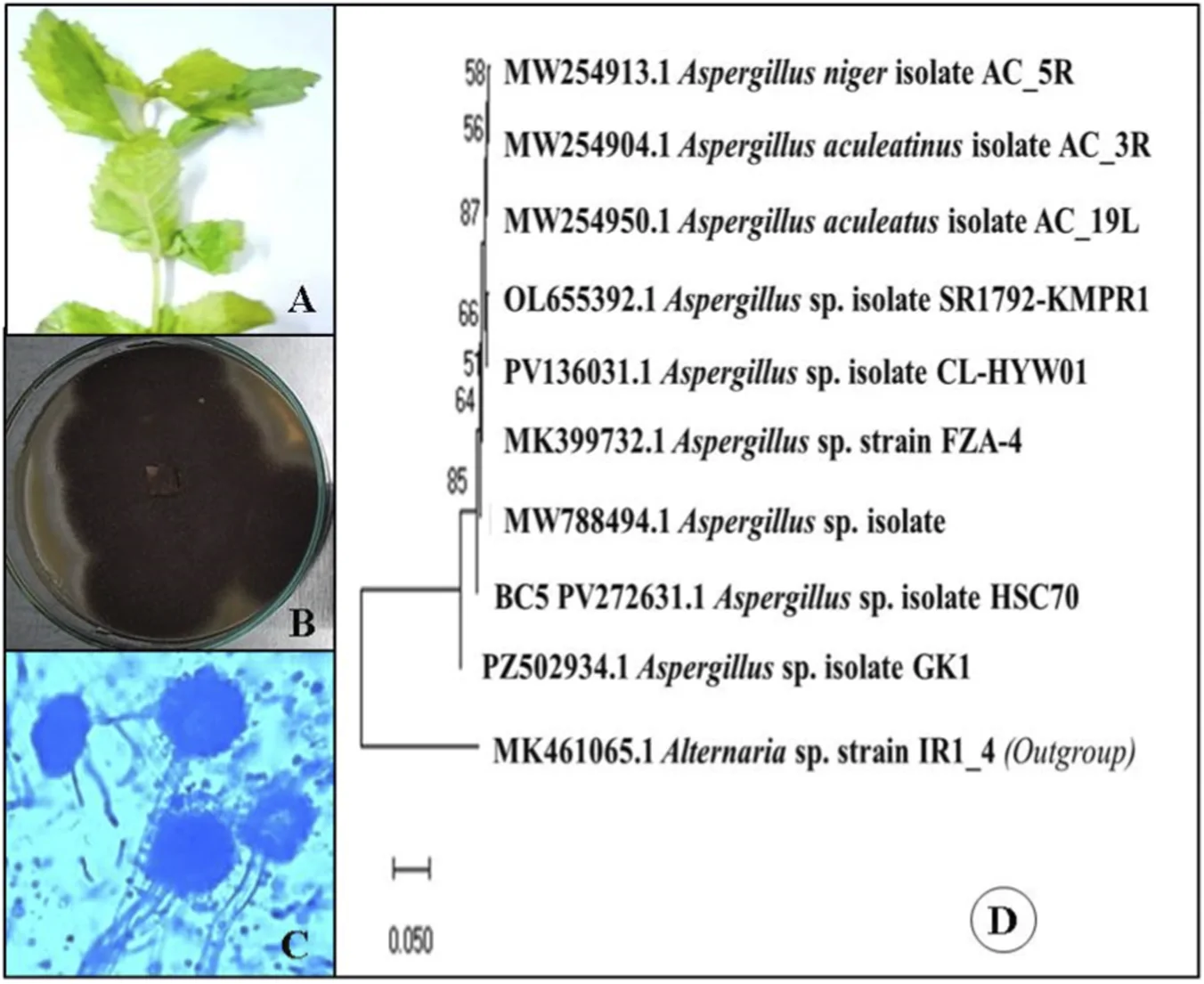
Isolation and identification of endophytic fungus from the roots of *M. spicata*. Here, **(A)** *M. spicata*; **(B)** Isolated fungi; **(C)** LPCB wet mounting at 40x; **(D)** phylogenetic tree of *Aspergillus* sp. isolate GK1 (GeneBank ID: PZ502934.1).

*Aspergillus* species are well known to be the endophytes of medicinal plants. Endophytic fungi belonging to the genus *Aspergillus* have been shown to grow in various host plants and to produce a wide range of secondary metabolites with structural diversity, such as alkaloids, terpenoids, polyketides, phenolic compounds, peptides, and other bioactive compounds that exhibit antimicrobial, antioxidant, anticancer, anti-inflammatory, and enzyme inhibitory properties (Kusari et al., 2009; Budhiraja et al., 2013; Morales-Sánchez et al., 2021). The identification of *Aspergillus* sp. isolate GK1 from *M. spicata* in the present study further supports these observations and highlights its potential source of natural products.

### 3.2 Antimicrobial activity of the fungal extract

The antibacterial potential of the extract was tested against the selected Gram-positive and Gram-negative bacterial pathogens using agar well diffusion method and the results are presented by the mean zone of inhibition (±SD) in Table 1 and Figure 2. The fungal extract exhibited measurable antibacterial activity against all the tested bacterial strains, whereas the negative control (dimethyl sulfoxide) showed no inhibitory effect, confirming that the observed activity was associated with the fungal extract under the conditions tested. The tested bacteria with the highest antibacterial activity were *E. coli* (15.66 + 0.57 mm) followed by *S. aureus* (14.66 + 1.52 mm), *Staphylococcus epidermidis* (14 + 0.00 mm) and *B. subtilis* (13.33 + 0.57 mm). The inhibition zones formed by the fungal extract were found to be less than that of the standard drug but still showed that the extract has antibacterial activities against both Gram-positive and Gram-negative bacteria. The positive control showed the remarkable antibacterial activity with the inhibition zone from 22.00 ± 1.00 mm to 26.00 ± 0.00 mm which indicates the efficiency of the assay. The findings suggest that the endophytic fungal extract has promising antibacterial activity under the experimental conditions and may warrant further investigation as a source of antimicrobial metabolites.

**FIGURE 2:**
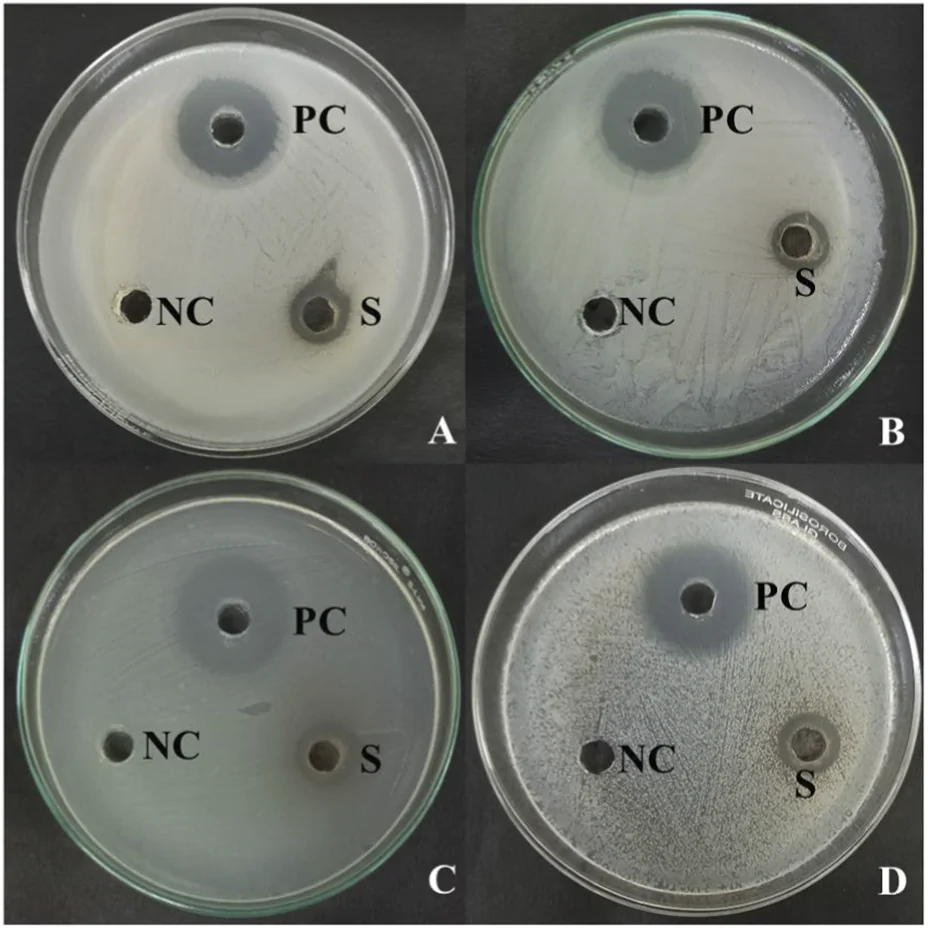
Antimicrobial activity of ethyl acetate extract of *Aspergillus* sp. isolate GK1 against clinical bacterial isolates. Here, **(A)** *S. aureus,* **(B)** *B. subtilis,* **(C)** *S. epidermidis,* and **(D)** *E. coli*. PC, Positive control (Gentamicin at 100 μg/mL); NC, Negative Control (DMSO); S, Sample (Extract at 1 mg/mL).

Further, the MIC and MBC were measured to confirm the antimicrobial activity of the extract. The minimum inhibitory concentration of the extract was the lowest against *E. coli* (16 μg/mL) followed by *S. aureus* (32 μg/mL) while the minimum inhibitory concentration of *S. epidermidis* and *B. subtilis* was 64 μg/mL. Likewise, the minimum bactericidal concentration values were 32, 32, 64 and 128 μg/mL against *E. coli*, *S. aureus*, *S. epidermidis* and *B. subtilis*, respectively. The MIC and MBC values are relatively low which suggests that the fungal extract has measurable bacteriostatic and bactericidal activity especially against *E. coli* and *S. aureus*. Susceptibility differences between the bacterial species could be due to differences in the cell wall structure, permeability, and intrinsic resistance mechanisms.

The present results are in line with earlier reports that have shown the antimicrobial properties of endophytic fungi. Mendieta-Brito et al. (2025) reported that ethyl acetate extracts of endophytic fungi isolated from the plants growing in Amazon forest showed good antibacterial activity between 15.6 and 500 μg/mL against *S. aureus, Enterococcus faecalis* and *Propionibacterium acnes*, in addition to showing good levels of antifungal activity against *Helminthosporium* sp.*, Fusarium oxysporum* and *F. solani*. Likewise, Sharma et al. (2025) extracted two anthraquinone derivatives (nigrosporin B and acetoxybostrycin) from the endophytic fungus *Nigrospora oryzae* which showed highly effective antibacterial activity against *S. aureus*, *Bacillus cereus*, and *E. coli*. Collectively, these studies, together with the present investigation, reinforce the growing evidence that endophytic fungi represent a rich reservoir of structurally diverse antimicrobial metabolites that may have potential for further development, including against multidrug-resistant bacterial pathogens, following isolation and evaluation of individual active compounds.

### 3.3 Chemical antioxidant and radical scavenging property

The ethyl acetate extract of *Aspergillus* sp. isolate GK1 was tested for chemical antioxidant capacity with various complementary *in vitro* assays such as DPPH radical scavenging, scavenging of hydrogen peroxide, reducing power and total antioxidant assays. Throughout all experiments ascorbic acid was used as an external positive control to set standard benchmarks. The Chemical antioxidant capacity of the endophytic fungal extract was highly significant (P < 0.05) and concentration dependent in all the experiments. The chemical antioxidant capacity showed a progressive increase, starting from 12.5% at 12.33% ± 0.96% at 12.5 μg/mL up to 65.19% ± 1.08% at 100 μg/mL in DPPH free radical scavenging assay with an IC50 value of 72.66 μg/mL (Figure 3A). Likewise, in the hydrogen peroxide-scavenging assay, the extract showed a concentration-dependent increase in H_2_O_2_-scavenging response, at a dose of 12.5 μg/mL, the scavenging metrics increased from 13.19% ± 0.62% to a maximum of 55.78% ± 1.31% inhibition in a dose of 100 μg/mL with IC50 of 86.30 μg/mL (Figure 3B). In addition, the extract was found to be reductive (Figure 3C) with high values in ferric-reducing power assay (OD at 400 μg/mL: 0.44 ± 0.01) and phosphomolybdenum total antioxidant capacity assay (OD at 400 μg/mL: 0.40 ± 0.008), with the chemical antioxidant property increasing with dose of the extract (Figure 3D). Collectively, these findings demonstrate chemical antioxidant capacity of the crude extract of *Aspergillus* sp. isolate GK1 extract in the tested biochemical assays and suggest the presence of reductive and hydrogen-donating metabolites, including potentially phenolic and flavonoid compounds. However, the contribution of individual metabolites cannot be established from these assays alone and requires bioassay-guided fractionation, isolation and structural characterization of the active constituents along with *in vivo* evaluation.

**FIGURE 3:**
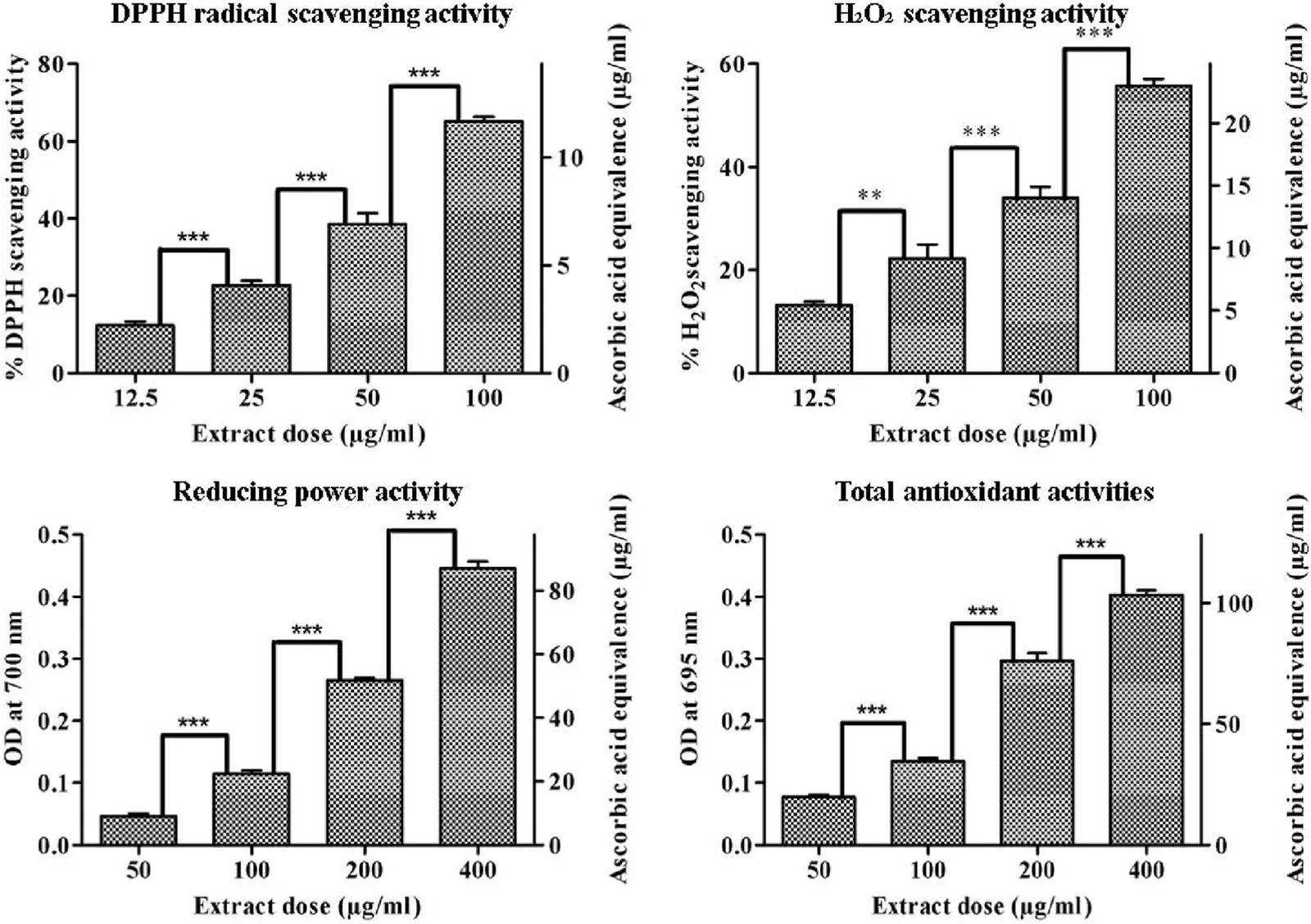
Chemical Antioxidant capacity of *Aspergillus* sp. isolate GK1 extract. Here, the fungal extract exhibited a significant (*p* < 0.05) dose-dependent increase in different chemical antioxidant assays.

The endophytic fungi of medicinal plants have been shown to be a good source of natural antioxidants through the production of various bioactive secondary metabolites in different studies. Yadav et al. (2014) reported that ethyl acetate extracts of endophytic fungi, including *Chaetomium* sp., *Aspergillus* sp., *A. peyronelii*, and *Aspergillus niger* isolated from *Eugenia jambolana*, exhibited remarkable chemical antioxidant capacity in DPPH radical scavenging, hydrogen peroxide scavenging, and reducing power assays. These chemical antioxidant potential was strongly positively correlated with total phenolic content, with extracts with 58–60 mg gallic acid equivalent/g having the highest antioxidant capacity suggesting that phenolic metabolites are major component in free radical scavenging. In parallel, Raunsai et al. (2018) found remarkable antioxidant capacity in the extracts of endophytic fungi isolated from medicinal plants from Central Sulawesi. The DPPH radical scavenging property of several isolates was attributed mainly to presence of phenolic, flavonoid and other secondary metabolites which are known to scavenge free radicles and this helps to reduce the oxidative stress. The results of present study are in accordance with the above findings further supporting the possible contribution of phenolic and flavonoid rich endophytic fungal metabolites in their chemical antioxidant capacity observed in the present biochemical assays.

### 3.4 Total phenolic and flavonoid content

Phenolic and flavonoid are very well known for their various bioactive properties, serving as essential antioxidants, immune system stimulators, antimicrobials and anti-inflammatory agents. In recent years, endophytic fungi of medicinal plants have proven to be a wealth of resources for the production of these types of secondary metabolites (Navarro García et al., 2025; Rao and Zheng, 2025). The quantitative analysis of the crude ethyl acetate extract of *Aspergillus* sp. isolate GK1 isolated from the roots of *M. spicata* showed that both phenolic and flavonoid compounds were present. The extraction yield showed high total phenolic content of 55.17 mg GAE/g of fungal extract and high total flavonoid content of 47.09 mg QE/g of fungal extracts. Moreover, the dose-response relationship was clearly shown in the experimental data, and it was statistically significant (P < 0.05). The amount of phenols and flavonoids detected increased gradually with the increase in the dose of the crude extract, ranging from 125 µg to 1000 µg (Figures 4A,B).

**FIGURE 4:**
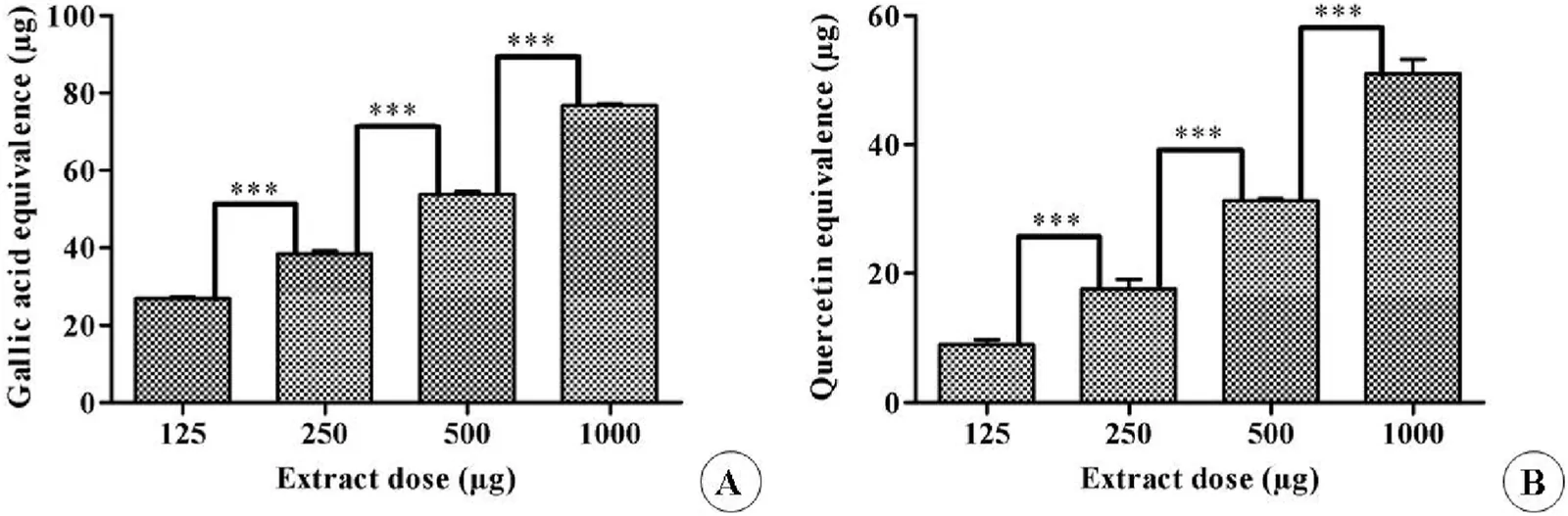
Total phenolic **(A)** and total flavonoid content **(B)** of ethyl acetate extract of *Aspergillus* sp. Isolate GK1. Here, the fungal extract exhibited a significant (*p* < 0.05) dose-dependent increase in TPC and TFC.

This concentration-dependent finding indicates that the crude extract contains measurable levels of phenolic and flavonoid compounds and may partly explain the chemical antioxidant properties observed in the present study. However, these assays do not establish that these compounds are solely responsible for the observed biological properties.

### 3.5 Relation between TPC, TFC and different chemical antioxidant activities

The crude extract of *Aspergillus* sp. isolate GK1 exhibited strong chemical antioxidant properties in multiple *in vitro* assays. Since crude fungal extracts contain a complex mixture of metabolites, it is not possible to attribute the observed chemical antioxidant properties to a single compound. Endophytic fungi produce a wide range of bioactive metabolites, including phenolics, flavonoids, alkaloids, polyketides, and polysaccharides, which may collectively contribute to the antioxidant effect. Although bioassay-guided isolation is required to identify the major active constituents, previous studies have shown that phenolic and flavonoid compounds are among the principal contributors to the antioxidant capacity of natural extracts. Therefore, correlation analysis between total phenolic content (TPC), total flavonoid content (TFC), and chemical antioxidant capacity was performed to examine their possible association with the observed antioxidant potential.

The correlation analysis demonstrated a strong positive relationship between the total phenolic content (TPC), total flavonoid content (TFC), and chemical antioxidant activities of the studied sample (Figure 5). The coefficient of determination (R^2^) values obtained from all regression analyses ranged from 0.9752 to 0.9986, indicating excellent linear correlations between the tested fungal metabolites and chemical antioxidant potential. These findings indicate that phenolic and flavonoids may be associated with the chemical antioxidant capacity of the samples.

**FIGURE 5:**
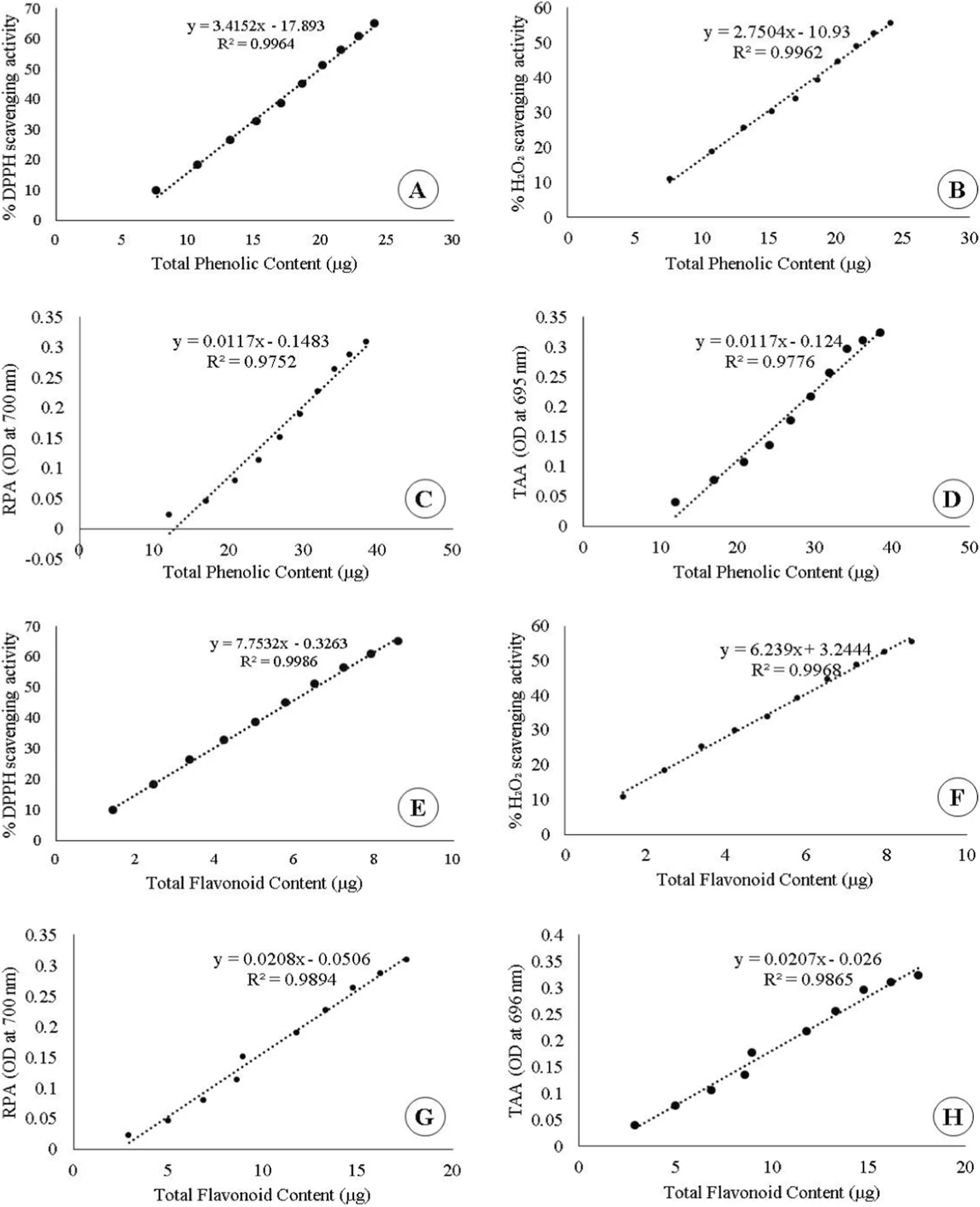
Correlation of total phenolic **(A–D)** and total flavonoid **(E–H)** contents with DPPH radical scavenging, H_2_O_2_ scavenging, reducing power, and total antioxidant activities respectively. Strong positive correlations were observed for all parameters.

The DPPH radical scavenging potential showed a good positive correlation with TPC (R^2^ = 0.9964), meaning that samples with higher phenolic content also showed greater free radical scavenging property. Likewise, an extremely significant positive correlation was observed between TPC and the scavenging property against hydrogen peroxide (H_2_O_2_) (R^2^ = 0.9962), indicating an association between phenolic content and H_2_O_2_ scavenging property. The RPA also showed an increase with the increasing phenolic content and was highly correlated (R^2^ = 0.9752), indicating that the phenolic constituents are good electron donors. In addition, the total antioxidant activity (TAA) showed a positive strong correlation with TPC (R^2^ = 0.9776), indicating an association between phenolic content and the chemical antioxidant capacity of the samples.

In the same manner, the results of the total flavonoid content revealed a high positive correlation with all chemical antioxidant assays. The correlation between TFC and DPPH radical scavenging property was very high (R^2^ = 0.9986), suggesting that the flavonoids content is strongly associated with the DPPH radical scavenging property. There was also a highly significant positive correlation between the TFC and H_2_O_2_ scavenging property (R^2^ = 0.9968), which indicates that flavonoids may contribute in scavenging of H_2_O_2_. The correlation between the reducing activity and TFC was significant (R^2^ = 0.9894) indicating that flavonoids may contribute to reducing activity and electron transfer ability. Similarly, TAA showed a significant correlation with TFC (R^2^ = 0.9865) indicating an association between flavonoid content and the total antioxidant activity.

Overall, the results indicate a strong positive association between TPC, TFC, and the chemical antioxidant property observed in the crude extract. The high R^2^ values suggest that phenolic and flavonoid compounds may be important contributors to chemical antioxidant capacity of the extract. However, as the extract contains a complex mixture of metabolites, the contribution of other bioactive constituents cannot be excluded. Therefore, while phenolics and flavonoids appear to play an important role in the chemical antioxidant potential of the studied samples, bioassay-guided isolation and characterization of the active compounds are required to confirm their individual contributions (Navarro García et al., 2025; Rao and Zheng, 2025).

### 3.6 *In vitro* cytotoxic activity against MCF-7 cell lines

The cytotoxicity of ethyl acetate extract of *Aspergillus* sp. isolate GK1 was assessed against MCF-7 breast cancer cell lines by using MTT assay. The extract showed statistically significant (P < 0.05) dose-response cytotoxic activity which gave an IC50 value of 69.20 μg/mL. After 24 h of treatment, a significant increase in percent cytotoxicity was seen, which increased from around 10% at 5 μg/mL to more than 50% at 80 μg/mL (Figure 6). These quantitative results were confirmed by the morphological changes observed from the examination of the MCF-7 cells treated with different concentrations of the extract and compared with the normal cellular morphology of the control group (Figures 6A–H). Exposure to higher concentrations produced specific changes in cell damage including progressive cell shrinkage, rounding, loss of contact inhibition and significant decrease in total viable cell number. The significant morphological alteration at higher extract concentrations is consistent with cytotoxic effects; however, these morphological observations alone do not establish the involvement of specific apoptotic pathways. Chlorpromazine was used as positive control and inhibited the cells with an IC50 value of 12.61 μg/mL.

**FIGURE 6:**
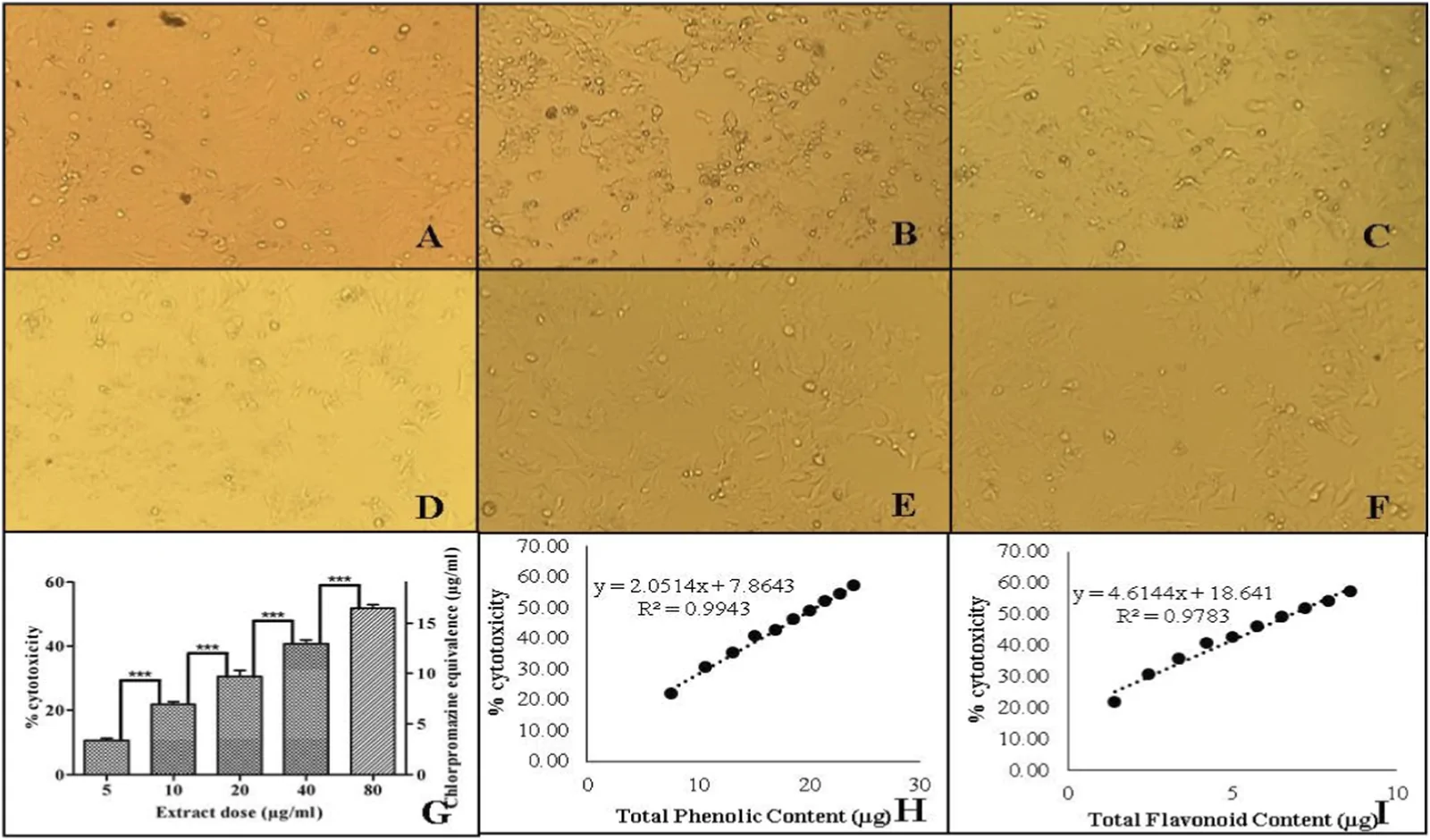
*In vitro* cytotoxic activity of the ethyl acetate extract of *Aspergillus* sp. isolate GK1 against MCF-7 cells. (**A–F)** show cell morphology after 24 h treatment with 0, 5, 10, 20, 40, and 80 μg/mL of the extract, respectively. **(G)** shows significantly (*P* < 0.05) dose-dependent cytotoxicity, while **(H, I)** depict the correlation of cytotoxicity with TPC and TFC, respectively.

The present findings are consistent with previous reports highlighting the cytotoxic potential of endophytic fungi. Nwobodo et al. (2024) have tested extracts of *Lasiodiplodia theobromae* and *Curvularia lunata* that were isolated from Nigerian medicinal plants and found that the extracts exhibited relatively low cytotoxic activity against Jurkat cell lines (CC_50_ > 100 μg/mL). Analysis by Gas Chromatography–Mass Spectrometry indicated a number of metabolites, such as 2,4-di-tert-butylphenol, hexadecanoic acid, oxalic acid and isobutyl nonyl ester, which were believed to be responsible for the biological activities observed. On the other hand, crude extracts of endophytic fungus species *Trichoderma*, *Penicillium* and *Fusarium* isolated from *Anethum graveolens* showed significant cytotoxic activity against HepG2 human hepatocellular carcinoma cells with 89%–92% growth inhibition at 50 μg/mL (El-Zehery and associates, 2024). These results were believed to be due to the wide range of antifungal secondary metabolites, which target multiple cellular components. The isolate *Aspergillus* sp. GK1 showed noticeable cytotoxic activity against MCF-7 Breast cancer cells with an IC_50_ value of 69.20 μg/mL, indicating measurable cytotoxic potential under the conditions tested. However, more research involving bioassay-guided isolation of the active constituents and mechanistic investigations, including apoptosis, cell cycle analysis, mitochondrial dysfunction, and other relevant molecular pathways, together with testing against appropriate non-cancerous cell models and *in vivo* evaluation, will be essential to establish their therapeutic potential.

### 3.7 Correlation of cytotoxic activity with TPC and TFC

Although, the crude ethyl acetate extract of the *Aspergillus* sp. isolate (GK1) showed measurable cytotoxic effect against MCF-7 cells, it should be noted that crude fungal extracts are a complex mixture of secondary metabolites with multiple bioactivities. So the observed cytotoxic effect cannot be solely attributed on any single class of compounds. In the past, several fungal metabolites like phenolic compounds, flavonoids, alkaloids, terpenoids, polyketides, fatty acids, peptides, and other secondary metabolites have been reported to exhibit cytotoxic activity by inducing apoptosis, generating ROS, mitochondrial dysfunction, cell cycle arrest, and inhibiting cancer cell proliferation (Evidente et al., 2014; Umer et al., 2026). Therefore, purification, characterization of the structure and biological evaluation of the individual active constituents are required to determine the main compounds responsible for the cytotoxic activity and to confirm their pharmacological potential.

Correlation analysis was carried out to explore the possible relation between the phenolic and flavonoid compounds with the cytotoxic activity of the fungal extract as in recent past several studies have identified these compounds with cytotoxic activity (Perveen and Al-Taweel, 2017; da Silva et al., 2025). The analysis showed that there were strong positive linear correlation between total phenolic content (R^2^ = 0.9943) and total flavonoid contents (R^2^ = 0.9783) with cytotoxic activity (Figures 6H,I). Based on these findings it may be suggested that higher TPC and TFC were associated with greater cytotoxic activity against MCF-7 cells. These correlations do not indicate a causal relationship between the two metabolites, but suggest that phenolic and flavonoid metabolites may be associated with the cytotixic activity of *Aspergillus* sp. isolate GK1, either singly, or in combination with other secondary metabolites. Further bioactivity-guided fractionation and mechanistic studies are needed to determine the individual compounds responsible for the observed cytotoxic effects.

### 3.8 Gas chromatography-mass spectrometry (GC-MS) analysis

Gas chromatography coupled with mass spectrometry (GC-MS) serves as a robust analytical standard for the comprehensive profiling of volatile and semi-volatile secondary metabolites in complex natural matrices. Endophytic fungi residing within the localized tissues of medicinal plants are well-documented to synthesize a broad spectrum of biologically and chemically active metabolites that often work synergistically with or mimic the metabolic pathways of their hosts.

In this study, the chemical profile of ethyl acetate crude extract of endophytic isolate of *Aspergillus* sp. GK1 isolated from roots of *M. spicata* was obtained by GC-MS. The resulting chromatogram revealed a complex chemical landscape characterized by several well-resolved peaks spanning across different retention times (Figure 7). The individual chemical entities were tentatively identified by comparing the mass fragmentation spectra with those in the National Institute of Standards and Technology (NIST) library.

**FIGURE 7:**
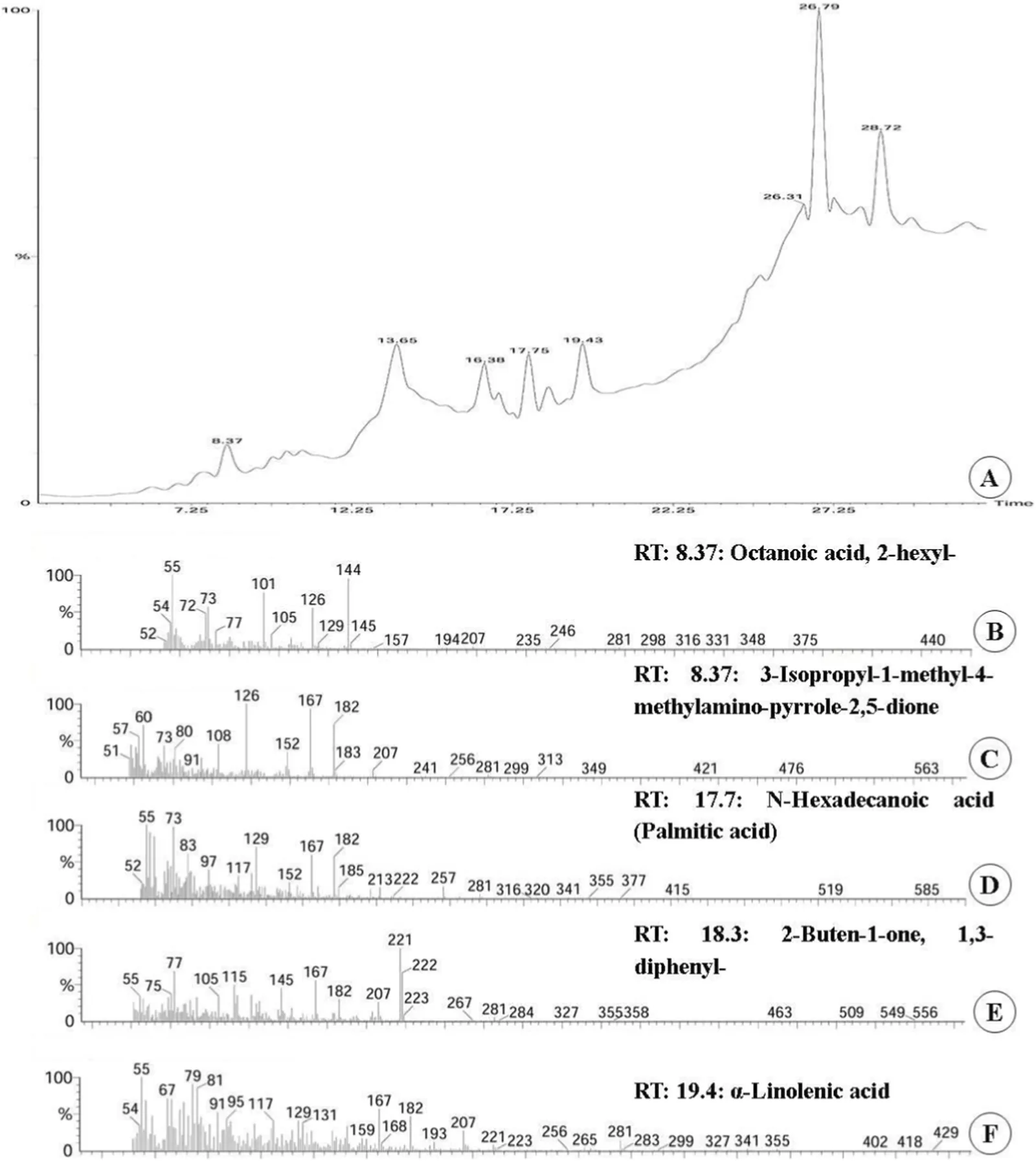
GC–MS analysis of the ethyl acetate extract of *Aspergillus* sp. isolate GK1 isolated from the roots of *Mentha spicata*. **(A)** shows the GC chromatogram, while **(B–F)** present the mass spectra of the major identified compounds: **(B)** Octanoic acid, **(C)** 3-Isopropyl-1-methyl-4-methylamino-pyrrole-2,5-dione, **(D)** N-Hexadecanoic acid (Palmitic acid), **(E)** 2-Buten-1-one, 1,3-diphenyl-, and **(F)** α-Linolenic acid.

A wide variety of metabolites, including Octanoic acid, 2-hexyl-, 3-Isopropyl-1-methyl-4-methylamino-pyrrole-2,5-dione, N-Hexadecanoic acid (Palmitic acid), 2-Buten-1-one, 1,3-diphenyl-, and α-Linolenic acid, were tentatively identified by GC-MS analysis. The mass spectra of the late eluting peaks at 26.79 and 28.72 min were dominated by fragment ions of siloxanes common with thermal column bleed. The characterization of fungal metabolites did not include these non-sample artifacts. The detailed mass spectrometric data including tentative identity, molecular formula, molecular weight and chemical classification is systematically tabulated in Table 2.

After a comprehensive literature survey, it was found that the metabolites obtained from the ethyl acetate extract of *Aspergillus* sp. isolate GK1 are reported to possess a wide range of potent biological activities against multiple targets. As detailed in Table 2, the primary constituents are heavily dominated by fatty acids, a heterocyclic pyrrole, and a chalcone framework, which are widely reported to exhibit antioxidant, anti-inflammatory, antimicrobial, and antifungal properties (Nowakowska, 2007; Özdemir et al., 2017; Zhuang et al., 2018). Compound like N-Hexadecanoic acid is also reported to possess cytotoxic and other biological activities (Zafaryab et al., 2019). α-linolenic acid is a well-known omega-3 fatty acid with reported anti-inflammatory and cardiovascular effects (Rajaram, 2014). However, the presence of these compounds in the present extract was based on GC-MS library matching, and their individual contribution to the observed biological activities cannot be established without purification and advance analytical experimentation. These studies provide a possible biochemical rationale for the observed antimicrobial, chemical antioxidant, and cytotoxic activities, but further compound-specific studies are required.

### 3.9 Molecular docking

To explore the binding potential of GC-MS–tentatively identified compounds from the endophytic fungal extract against selected antibacterial, cancer-associated, and oxidative stress–related protein targets, molecular docking analysis was performed. The docking results as binding free energy and predicted inhibition constant (pKi) are given in Tables 3–5, and the two-dimensional protein-ligand interaction maps are shown in Figure 8. Overall, when the binding free energy is more negative, it implies a predicted stronger binding interaction between the ligand-receptor complex. Of the compounds screened, CID 95329 showed the most favorable predicted docking scores among the compounds evaluated across the selected targets and was therefore selected for exploratory computational follow-up.

**TABLE 3:** Binding affinities and predicted pKi values of GC–MS-identified metabolites docked against antibacterial recepto**r**.

6F86 (DNA GYRASE)			3VSL (PBP)		
Ligand ID	Binding free energy (kcal/mol)	pKi	Ligand ID	Binding free energy (kcal/mol)	pKi
CID 95329	−6.6	4.84	CID 95329	−7.2	5.28
CID 109035	−5.7	4.18	CID 5280934	−5.4	3.96
CID 5280934	−5	3.67	CID 597587	−4.9	3.59
CID 597587	−4.9	3.59	CID 109035	−4.8	3.52
CID 985	−4.5	3.3	CID 985	−4.6	3.37

**TABLE 4:** Binding affinities and predicted pKi values of GC–MS-identified metabolites docked against cancer associated receptor.

1JNX (BRCA 1)			1M17 (EGFR)		
Ligand ID	Binding free energy (kcal/mol)	pKi	Ligand ID	Binding free energy (kcal/mol)	pKi
CID 95329	−6.2	4.55	CID 95329	−7.6	5.57
CID 985	−4.1	3.01	CID 5280934	−5.6	4.11
CID 597587	−4	2.93	CID 597587	−5.2	3.81
CID 109035	−3.9	2.86	CID 109035	−5.2	3.81
CID 5280934	−3.7	2.71	CID 985	−5.1	3.74

**TABLE 5:** Binding affinities and predicted pKi values of GC–MS-identified metabolites docked against oxidative stress-associated recepto**r**.

1HD2 (human peroxiredoxin 5)			2CDU NAD(P)H oxidase (*L. sanfranciscensis*)		
Ligand ID	Binding free energy (kcal/mol)	pKi	Ligand ID	Binding free energy (kcal/mol)	pKi
CID 95329	−6.2	4.55	CID 95329	−9.1	6.67
CID 597587	−4.7	3.45	CID 5280934	−7.3	5.35
CID 109035	−4.3	3.15	CID 985	−6.5	4.77
CID 5280934	−4.3	3.15	CID 109035	−6.4	4.69
CID 985	−4	2.93	CID 597587	−5.5	4.03

**FIGURE 8:**
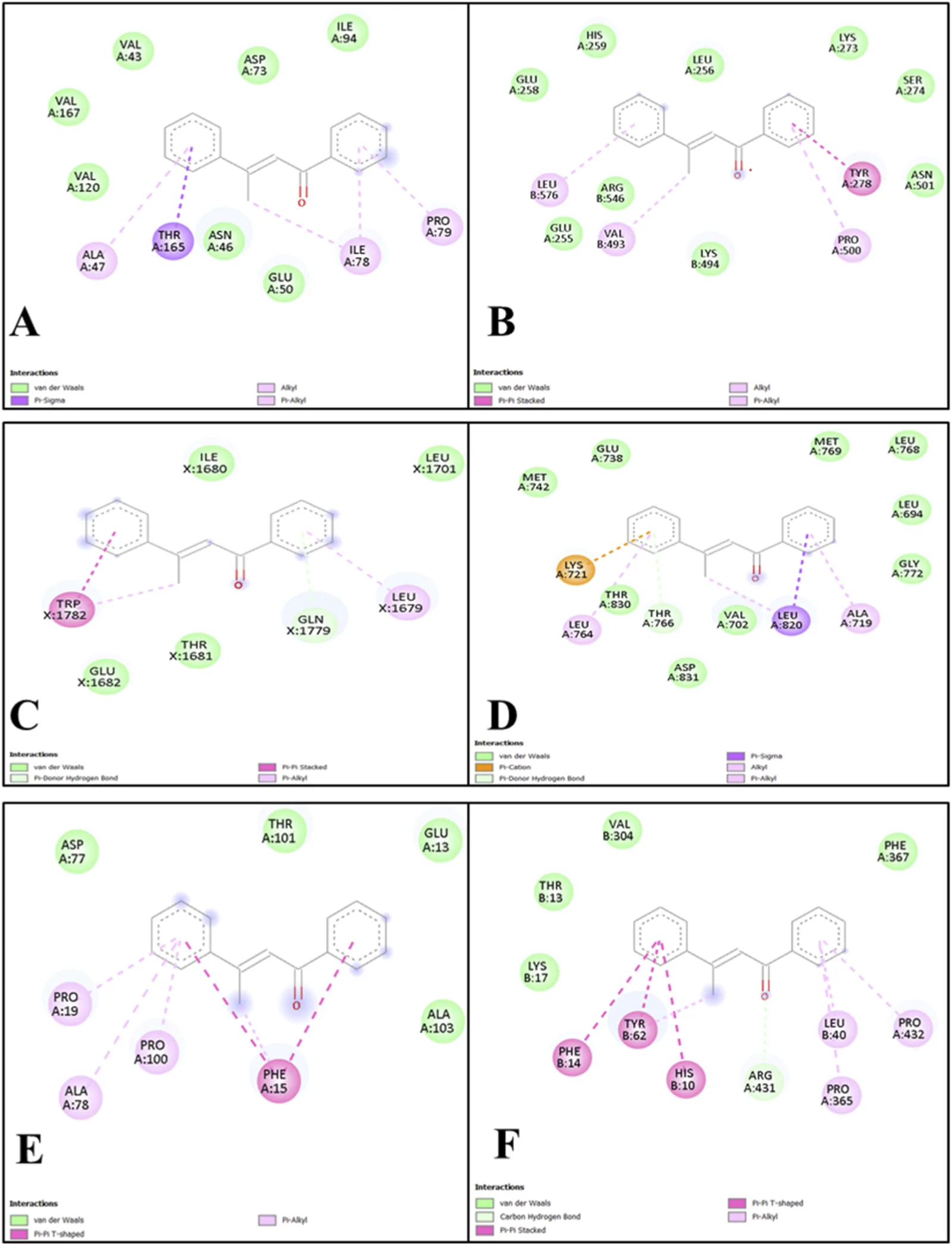
Two-dimensional (2D) interaction diagrams illustrating the binding interactions of the selected metabolites with the target proteins: **(A)** DNA Gyrase B (PDB ID: 6F86), **(B)** Penicillin-Binding Protein (PBP; PDB ID: 3VSL), **(C)** BRCA1 (PDB ID: 1JNX), **(D)** Epidermal Growth Factor Receptor (EGFR) Tyrosine Kinase (PDB ID: 1M17), **(E)** Human Peroxiredoxin 5 (PDB ID: 1HD2), and **(F)** NAD(P)H oxidase (NOX; PDB ID: 2CDU). The diagrams depict key protein–ligand interactions, including hydrogen bonds, π–π interactions, π–alkyl interactions, π–sigma interactions, carbon–hydrogen bonds, and van der Waals contacts.

CID 95329 bound to DNA Gyrase B (PDB ID: 6F86) and Penicillin-Binding Protein (PDB ID: 3VSL) with binding free energies of −6.6 kcal/mol and −7.2 kcal/mol, respectively, against the antibacterial targets, which corresponds to predicted pKi of 4.84 and 5.28 respectively (Table 3). The other metabolites had relatively lower affinities with binding energies of −4.5 to −5.7 kcal/mol and −4.6 to −5.4 kcal/mol to DNA Gyrase B and Penicillin-Binding Protein respectively. As the DNA gyrase is involved in bacterial DNA supercoiling and Penicillin-Binding Proteins in the biosynthesis of peptidoglycan, disabling these enzymes can theoretically interfere with bacterial survival and growth. Other studies using GC-MS-obtained metabolites have demonstrated that compounds with docking scores of about −6 to −8 kcal/mol against DNA gyrase have potential antibacterial effects and should be subjected to other biological tests (Chatterjee et al., 2023; Galma et al., 2021). Notably, although no binding site was specified, the unbiased search placed CID 95329 in the ATP-binding pocket of GyrB, interacting with Asn46, Asp73, Ile78, Pro79, Val120 and Thr165. This convergence on the functional ATPase site suggests possible ATP-competitive binding, a hypothesis consistent with the bactericidal profile of the extract (MBC/MIC ≤ 4).

Further enhanced performance of CID 95329 was further demonstrated by docking in the presence of the cancer associated receptors. The compound showed binding energies of −6.2 kcal/mol in BRCA 1 (PDB ID: 1JNX) and −7.6 kcal/mol in EGFR Tyrosine Kinase (PDB ID: 1M17), which is higher than the binding energies of the rest of the identified metabolites (Table 4). EGFR plays a key role in cell proliferation and cell survival signaling pathways, and BRCA 1 is implicated in the repair and maintenance of genomic stability. Similarly, CID 95329 localised within the ATP-binding cleft of EGFR. There it contacted the hinge residue Met769, the gatekeeper Thr766, and Lys721, Glu738 and Asp831, which together define the erlotinib-binding site of this structure (Stamos et al., 2002). Since chalcone-type α,β-unsaturated ketones can display promiscuous reactivity, and the identity of CID 95329 rests only on NIST library matching, these results are treated strictly as hypothesis-generating. Moreover, CID 95329 was the only aromatic, planar molecule among the GC–MS hits, and docking scoring functions tend to favour such rigid aromatic ligands. EGFR docking was included solely to ask whether the metabolite can structurally occupy a cancer-relevant kinase site. It is not presented as evidence of anticancer activity, which would require mechanistic studies and selectivity testing against normal cells. Similar studies of recent GC-MS-guided molecular docking studies have also reported that metabolites that can form stable complexes with EGFR can be utilized as promising lead molecules for further experimental investigation (Sruthy et al., 2025).

Among all receptors studied, the oxidative stress-associated targets yielded the best docking effects. CID 95329 bound human peroxiredoxin 5 (PDB ID: 1HD2) with a binding free energy of −6.2 kcal/mol and predicted pKi value of 4.55 and NADPH the *L. sanfranciscensis* NAD(P)H oxidase (PDB ID: 2CDU) with a binding free energy of −9.1 kcal/mol and predicted pKi value of 6.67 (Table 5). This is the best ligand protein interaction that has been recorded in the current study. This bacterial enzyme is an FAD-dependent oxidoreductase. As the docked pose occupied the FAD-binding region vacated during receptor preparation, this binding energy should be interpreted with caution and is not extrapolated to human NOX isoforms, and Peroxiredoxin 5 is an endogenous antioxidant enzyme that neutralizes peroxides and defends the cells against oxidative stress. The high docking score obtained relative to NADPH oxidase suggests a strong interaction of CID 95329 with an oxidative-stress-related target; however, it does not by itself demonstrate antioxidant activity or regulation of oxidative stress pathways in cells and need to be validated further. Similar analyses of natural products have also shown positive interactions of metabolites with NADPH oxidase and peroxiredoxin targets, and can support their potential involvement in antioxidant activity (Aboudia et al., 2025; Amara et al., 2025).

Figure 8 shows the two-dimensional interaction diagrams that further support the docking scores observed. Hydrophobic interactions that include π–alkyl, π–π stacked, π–π T-shaped, and π–σ contacts as well as extensive van der Waals contacts tended to stabilize the ligand–protein complexes. Furthermore, some of the complexes, especially with EGFR and NADPH oxidase, were found to have carbon-hydrogen bonds and conventional hydrogen bonds, which indicated predicted stabilization within the binding sites. These non-covalent interactions may contribute to the predicted binding affinity and specificity of ligand–protein complexes.

Overall, the molecular docking results indicated that CID 95329 exhibited comparatively stronger predicted binding across the selected bacterial, cancer-associated, and oxidative stress–related protein targets than the other compounds. Its highest affinity to NADPH oxidase (−9.1 kcal/mol), and next, EGFR (−7.6 kcal/mol) and Penicillin-Binding Protein (−7.2 kcal/mol) and the high number of stabilizing interactions shown in Figure 8 identify CID 95329 as a prioritized candidate for further experimental investigation. Molecular docking could only provide the predictive indication of the affinity between the ligand and the protein, and therefore these results should not be interpreted as direct signal of biological activity or target inhibition. The current results provide hypotheses that require further evaluation through purified-compound assays, target-specific experiments, and cellular studies.

### 3.10 ADMET study

In silico ADMET profiling provided computational predictions of absorption, distribution, metabolism and toxicity-related endpoints for CID 95329. These predictions are model-dependent and should be interpreted only as preliminary computational descriptors. They do not establish pharmacokinetic behavior, safety, or therapeutic suitability in biological systems. In the current study ADMET profile of CID 95329 was analyzed through pkCSM server while the predicted ADMET parameters are indicated in Table 6. The compound showed a high intestinal absorption (96.95) and Caco-2 permeability (1.366log Papp) which predicted favorable intestinal absorption characteristics. Caco-2 permeability has become a predictive parameter of intestinal absorption of drugs, and its values tend to be higher with efficient results in terms of membrane transportation and bioavailability (Awortwe et al., 2014). Aglthough the compound has a fairly low water solubility (log mol/L = −4.632), it is not expected to be either a substrate or inhibitor of P-glycoprotein, which means may indicate a lower likelihood of P-glycoprotein-mediated efflux. The profile of distribution showed that it had moderate level of distribution (0.331log L/kg) and it can cross the blood-brain barrier (0.541log BB), which predicts potential distribution to the central nervous system. Other comparable studies of ADMET have found that compounds with BBB permeability and desirable distribution properties can have increased therapeutic effects based on the site of interest (Rushendran and Chitra, 2024).

**TABLE 6:** Predicted ADMET properties of the computationally prioritized compound CID 95329 using the pkCSM online server.

Property	Model name	Predicted value	Unit
Absorption	Water solubility	−4.632	Numeric (log mol/L)
	Caco2 permeability	1.366	Numeric (log Papp in 10^−6^ cm/s)
	Intestinal absorption (human)	96.948	Numeric (% absorbed)
	Skin permeability	−2.195	Numeric (log Kp)
	P-glycoprotein substrate	No	Categorical (yes/no)
	P-glycoprotein I inhibitor	No	Categorical (yes/no)
	P-glycoprotein II inhibitor	No	Categorical (yes/no)
Distribution	VDss (human)	0.331	Numeric (log L/kg)
	Fraction unbound (human)	0	Numeric (Fu)
	BBB permeability	0.541	Numeric (log BB)
	CNS permeability	−1.313	Numeric (log PS)
Metabolism	CYP2D6 substrate	No	Categorical (yes/no)
	CYP3A4 substrate	Yes	Categorical (yes/no)
	CYP1A2 inhibitior	Yes	Categorical (yes/no)
	CYP2C19 inhibitior	Yes	Categorical (yes/no)
	CYP2C9 inhibitior	Yes	Categorical (yes/no)
	CYP2D6 inhibitior	No	Categorical (yes/no)
	CYP3A4 inhibitior	No	Categorical (yes/no)
Excretion	Total clearance	0.247	Numeric (log mL/min/kg)
	Renal OCT2 substrate	No	Categorical (yes/no)
Toxicity	AMES toxicity	No	Categorical (yes/no)
	Max. tolerated dose (human)	0.99	Numeric (log mg/kg/day)
	hERG I inhibitor	No	Categorical (yes/no)
	hERG II inhibitor	No	Categorical (yes/no)
	Oral rat acute toxicity (LD50)	1.978	Numeric (mol/kg)
	Oral rat chronic toxicity (LOAEL)	1.208	Numeric (log mg/kg_bw/day)
	Hepatotoxicity	No	Categorical (yes/no)
	Skin sensitisation	Yes	Categorical (yes/no)
	*T. pyriformis* toxicity	1.201	Numeric (log ug/L)
	Minnow toxicity	0.339	Numeric (log mM)

According to the metabolic predictions, CID 95329 is a CYP3A4 substrate and an inhibitor of CYP1A2, CYP2C19, and CYP2C9, which means that it might be involved in the hepatic metabolism and could have the potential for drug–drug interactions. Nevertheless, the lack of inhibition of CYP2D6 and CYP3A4 may reduce the predicted risk of inhibition through these pathways. It has been demonstrated previously that substances that react with cytochrome P450 enzymes could have complicated metabolic behavior, which needs to be confirmed in an experiment (Prasetyawan et al., 2025). CID 95329 was predicted to be non-mutagenic (AMES negative), non-hepatotoxic, and non-inhibitory against hERG I and II channels, which suggests a low predicted risk for these endpoints. It is especially desirable that the compounds do not interact with hERG, since these interactions are typically accompanied by adverse cardiac effects. These results are related to the earlier works in the field of ADMET-based studies, in which non-mutagenic and non-hepatotoxic metabolites were deemed safe and promising drug candidates (Taskin et al., 2021).

Nonetheless, this sensitization prediction of skin indicates that some caution might be necessary in the formulation when it involves the dermal exposure. On balance, the ADMET analysis provides preliminary *in silico* indication of favorable absorption and distribution characteristics and relatively low predicted toxicity for CID 95329; however, these predictions cannot be considered evidence of clinical safety or therapeutic suitability. The combination of these findings and its good performance in molecular docking supports the prioritization of CID 95329 for future experimental analysis, including isolation, structural confirmation, compound-specific biological assays, pharmacokinetic studies, and toxicity evaluation.

### 3.11 Study limitations and future perspectives

The present study represents an initial investigation of the biological and chemical characteristics of the crude ethyl acetate extract of *Aspergillus* sp. GK1. In view of this we would like to acknowledge few limitations of this study. First, the observed biological activities were evaluated using the crude extract, and therefore the specific metabolites responsible for these effects could not be definitively identified. Although GC–MS analysis tentatively identified several compounds and molecular docking prioritized potential lead molecules, these computational predictions require further experimental analysis following bioassay-guided isolation and structural characterization of the active constituents. Furthermore, the fungal isolate was identified at the genus level based on ITS sequencing, and species-level identification of black *Aspergillus* isolates would require additional phylogenetic markers such as calmodulin, β-tubulin, or RPB2. The cytotoxic activity was evaluated against the MCF-7 breast cancer cell line, and no normal cell lines were included to determine the selectivity index. Moreover, targeting cancer cells was limited to cytotoxicity evaluation, and the underlying molecular mechanisms were not investigated. Finally, all biological properties were assessed *in vitro*, and *in vivo* studies are required to establish the efficacy, safety, pharmacokinetic properties, and therapeutic relevance of the identified bioactive metabolites.

Future studies should focus on bioassay-guided fractionation, isolation and structural characterization of the active constituents, confirmation of their identities using complementary analytical techniques (e.g., LC–MS/MS and NMR), evaluation of the biological activities of purified compounds, elucidation of their molecular mechanisms of action, and analysis through appropriate *in vitro* and *in vivo* studies. In addition, optimization of fermentation conditions, metabolomic profiling, and genomic analysis may facilitate enhanced metabolite production and the discovery of novel bioactive compounds. Such comprehensive studies will be necessary before considering any individual metabolite or *Aspergillus* sp. GK1-derived products as potential therapeutic agents.

## 4 Conclusion

Based on the present study, the endophytic isolate of *Aspergillus* sp. GK1, isolated from the root of *M. spicata* represents a potential source of biologically active secondary metabolites. The crude ethyl acetate extract exhibited antibacterial activity against the tested Gram-positive and Gram-negative bacterial strains, concentration-dependent chemical antioxidant property, and cytotoxicity against MCF-7 breast cancer cells. The total phenolic and flavonoid contents showed a strong positive correlation with the chemical antioxidant and cytotoxic activities, suggesting that these metabolites may contribute to the observed bioactivities, although the specific active compounds remain to be identified. GC-MS analysis tentatively identified several metabolites in the crude extract, while molecular docking predicted that 2-Buten-1-one, 1,3-diphenyl- exhibited favorable binding affinities against the selected antibacterial, cancer-associated, and oxidative stress–related protein targets. These docking results provide a computational indication of possible molecular interactions but do not establish biological activity or direct target engagement. The ADMET analysis further provided preliminary *in silico* information regarding the predicted pharmacokinetic and toxicity properties of the selected compound. Collectively, these findings provide a preliminary characterization of the antibacterial, cytotoxic, and chemical antioxidant properties of the *Aspergillus* sp. GK1 extract, together with exploratory computational analyses of selected tentatively identified metabolites. The results highlight the potential of endophytic fungi associated with medicinal plants as sources of chemically diverse metabolites. Nevertheless, comprehensive analysis through bioassay-guided isolation, structural characterization, mechanistic investigations, appropriate selectivity studies using normal cells and *in vitro* and *in vivo* studies will be essential before individual metabolites or *Aspergillus* sp. GK1 derived products can be considered potential therapeutic candidates.

## Data Availability

The datasets presented in this study can be found in online repositories. The names of the repository/repositories and accession number(s) can be found in the article/Supplementary Material.
