# Supplementary material for: Integrated in vitro and in silico assessment of antimicrobial, chemical antioxidant, and cytotoxic activities of endophytic Aspergillus sp. GK1 isolated from Mentha spicata roots
Source: Front Pharmacol. 2026 Oct 16;17:1937405. doi: 10.3389/fphar.2026.1937405 (PMC13719591; doi:10.3389/fphar.2026.1937405)
Supplement: Supplementary file 3 [file DataSheet3.pdf]

ConPhyMP checklist of items for conducting and reporting analytical methods[1,2] relevant for extract type C (for species or botanical drugs derived from plants not widely used or traded)

SECTION/TOPIC	ITEM NO.	CHECKLIST ITEM	YES	NO	NOT APPLICABLE	PAGE NO., IF ANY
Type of extract	1	C – Confirm that the species or botanical drug under investigation is not a widely used or traded one.	✓			The investiga
Alternative methods for extract characterisation/chemical analysis	3	(a) Single chemical fingerprinting method with three different detection parameters (i.e., altered detection parameters, like TLC/HPTLC with different staining reagents and/or UV excitation wavelengths, HPLC-DAD/LC-DAD with different wavelengths). The same applies to coupling MS or NMR to chromatographic techniques.	✓			8, 22-24
		(b) No descripton of marker substances is needed but may be provided.			✓	Identificatior
Use of reference standards	4	(a) Direct overlay of the chromatogram of the sample with that of an officially specified reference standard (if applicable)			✓	Identificatior
		(b) Chromatographic fingerprinting: Direct overlay of the chromatogram of the sample with that of official reference standards of the powdered plant material or the dry extract from the plant material.			✓	Identificatior
Comparison of different extracts/ samples of the same plants	5	(a) Direct comparison of the chromatographic/spectroscopic system and/or scoring system for "similarity" to be followed.	✓			22-24 (Identi

Note: Please also include here the following information about your submitted manuscript:

Name of the journal:	Frontiers in Pharmacology - Ethnopharmacology
Date of the enquiry:	5 August 2026
Title of the manuscript:	Integrated In vitro and In silico Assessment of Antimicrobia
List of the authors:	Fahad Almarshadi , Walid Alesefir, Gaurav Kumar

[1] Please acknowledge/cite this as follows: Heinrich M, Jalil B, Abdel-Tawab M, Echeverria J, Kulić Ž, McGaw LJ, et al. Best Practice in the chemical characterisation of extracts used in pharmacological and toxicological research—The ConPhyMP—Guidelines. Frontiers in Pharmacology. 2022;13:953205. https://doi.org/10.3389/fphar.2022.953205

[2] We strongly recommend reading this checklist in conjuncton with ConPhyMP 2022 explanaton and elaboraton for important clarifications on all items. If relevant, we also recommend after reading Heinrich et al. (2020) Best practice in research—Overcoming common challenges in phytopharmacological research. Journal of Ethnopharmacology. 2020;246:112230. https://doi.org/10.1016/j.jep.2019.112230
