# Supplementary material for: Integrated in vitro and in silico assessment of antimicrobial, chemical antioxidant, and cytotoxic activities of endophytic Aspergillus sp. GK1 isolated from Mentha spicata roots
Source: Front Pharmacol. 2026 Oct 16;17:1937405. doi: 10.3389/fphar.2026.1937405 (PMC13719591; doi:10.3389/fphar.2026.1937405)
Supplement: Supplementary file 4 [file Table1.docx]

**Supplementary files**

**Supplementary Table S1: Characterization of clinical isolates of test bacteria used in this study**

| **Characteristic** | ***Escherichia coli*** | ***Staphylococcus aureus*** | ***Staphylococcus epidermidis*** | ***Bacillus subtilis*** |
| --- | --- | --- | --- | --- |
| **Growth on nutrient agar** | Smooth, circular, moist, cream-colored | Golden-yellow, smooth, convex | White, smooth, convex | Large, rough, irregular, dry colonies |
| **Growth on MacConkey agar** | Positive (Pink colonies) | No growth | No growth | No growth |
| **Gram reaction** | Gram-negative rods | Gram-positive cocci in clusters | Gram-positive cocci in clusters | Gram-positive rods |
| **Endospore** | Absent | Absent | Absent | Present |
| **Motility** | Positive | Negative | Negative | Positive |
| **Catalase test** | Positive | Positive | Positive | Positive |
| **Oxidase test** | Negative | Negative | Negative | Negative |
| **Coagulase test** | Negative | Positive | Negative | Negative |
| **Indole test** | Positive | Negative | Negative | Negative |
| **Methyl Red (MR)** | Positive | Negative | Negative | Negative |
| **Voges–Proskauer (VP)** | Negative | Positive | Positive | Positive |
| **Citrate utilization** | Negative | Negative | Negative | Positive |
| **Urease test** | Negative | Positive (weak/variable) | Positive | Negative |
| **Hemolysis on Blood Agar** | Usually γ (non-hemolytic) | β-hemolytic | γ-hemolytic | Usually β-hemolytic or variable |
| **Mannitol fermentation** | Positive | Positive | Negative | Negative |

**Supplementary Table S2: Antibiotic Sensitivity profile of the test bacteria used in this study**

| **Antibiotic** | **Disc Code** | **Disc Potency (µg/disc)** | ***Escherichia coli*** | ***Staphylococcus aureus*** | ***Staphylococcus epidermidis*** | ***Bacillus subtilis*** |
| --- | --- | --- | --- | --- | --- | --- |
| Ampicillin | AMP | 10 | R | R | R | S |
| Cefoxitin | FOX | 30 | S | S | R | S |
| Ciprofloxacin | CIP | 5 | S | S | S | S |
| Gentamicin | GEN | 10 | S | S | S | S |
| Tetracycline | TE | 30 | S | S | I | S |
| Chloramphenicol | C | 30 | S | S | S | S |
| Erythromycin | E | 15 | R | S | R | S |
| Vancomycin | VA | 30 | R | S | S | S |
| Levofloxacin | LEV | 5 | S | S | S | S |
| Kanamycin | K | 30 | S | S | S | S |
| Amikacin | AK | 30 | S | S | S | S |
| Azithromycin | AZM | 15 | R | S | I | S |

S = Sensitive, I = Intermediate, R = Resistant

**Supplementary Table S3:** Grid box parameters used for blind (whole-receptor) molecular docking in AutoDock Vina.

| **Target category** | **Receptor (PDB ID)** | **Grid centre (Å)** | | | **Grid size (Å)** | | |
| --- | --- | --- | --- | --- | --- | --- | --- |
|  |  | **x** | **y** | **z** | **x** | **y** | **z** |
| Antibacterial | DNA gyrase B (6F86) | 67.315 | 31.922 | 54.435 | 50 | 60 | 62 |
|  | Penicillin-binding protein (3VSL) | 34.669 | -38.942 | 6.499 | 92 | 134 | 97 |
| Cancer-associated | BRCA1 BRCT domain (1JNX) | 24.661 | 58.646 | 37.047 | 75 | 67 | 46 |
|  | EGFR tyrosine kinase (1M17) | 23.296 | 9.895 | 59.387 | 107 | 80 | 66 |
| Oxidative stress-associated | Human peroxiredoxin 5 (1HD2) | 7.936 | 42.687 | 20.122 | 51 | 50 | 57 |
|  | NAD(P)H oxidase, L. sanfranciscensis (2CDU) | 10.740 | 4.980 | 25.795 | 80 | 84 | 114 |

Grid boxes were set to enclose the entire receptor structure so that no binding site was pre-defined. Docking was performed in AutoDock Vina with exhaustiveness = [8], and the lowest-energy pose was selected for interaction analysis.
